# Purchasing Primary Care Services for Quality Chronic Care: Capitation With Performance Payments in Four Countries

**DOI:** 10.1002/hpm.3929

**Published:** 2025-04-08

**Authors:** Sarah L. Barber, Inke Mathauer, Megumi Rosenberg, Nicolas Larrain, Yunguo Liu, Qian Long, Anja Smith, Stevie Ardianto Nappoe, Luca Lorenzoni

**Affiliations:** ^1^ Centre for Health Development World Health Organization Kobe Japan; ^2^ Health Governance and Financing Department World Health Organization Geneva Switzerland; ^3^ Health Division Organisation for Economic Cooperation and Development Paris France; ^4^ Global Health Research Center Duke Kunshan University Jiangsu China; ^5^ Department of Economics Stellenbosch University Stellenbosch South Africa; ^6^ University of Gadjah Mada Yogyakarta Indonesia

**Keywords:** capitation, chronic care, health care, payment methods, performance payment, purchasing, quality

## Abstract

Improving quality of care for chronic conditions is central to addressing the large burden of premature mortality from non‐communicable diseases. This paper presents the main results from case studies of health care purchasing arrangements in Chile, China, Indonesia and South Africa, which involved paying providers of primary care services using capitation with performance pay to improve service quality and health outcomes for chronic conditions. In all four settings, changes to payment methods were accompanied by other enabling interventions to provide incentives to deliver health services in a better way, such as training and non‐financial incentives. However, the incentives in these programs were insufficient to drive significant changes in provider behaviour needed to improve quality. Design and implementation challenges included voluntary enrolment, quality metrics, performance targets, risk adjustment, payment certainty and levels, and withholding payments. The design and implementation challenges contributed to low patient volume or provider programme uptake resulting in lower‐than‐expected effects. The findings from this analysis underscore the importance of adjusting quality measures for patient health risk and complexity to avoid penalising health care providers for accepting patients with higher health risks. Relative or progressive quality targets may be more appropriate where wide diversity in providers' capacities exist, particularly in national programs, and may be used to encourage gradual quality improvements over time. Uncertainty about timing and levels of payment may have also undermined impact. Withholding performance payments as a penalty may reduce resources for quality improvements in these settings.

1

**Summary**

- Capitation with performance pay was used to improve chronic care quality.
- Four countries' experiences had varied implementation success.
- Performance pay alone did not drive significant provider behaviour changes.
- Adjusting quality measures for patient and social risk is crucial.

## 2 Introduction

Premature mortality among 30‐ to 70‐year‐olds account for 42% of all non‐communicable disease‐related deaths globally and is largely amenable to quality health care [1]. One means to improve quality is to link some of the payment to healthcare providers to the quality of care provided or patient health outcomes [2]. Systematic reviews to date report that performance payments have mixed or weak effects on quality and health outcomes for chronic conditions [3, 4, 5]. Substantial heterogeneity is described in the design, implementation, measurement and evaluation of payment models. It is unclear, however, whether such differences can account for lower‐than‐expected effectiveness [6].

Capitation payments are commonly used for primary care services. Payments are made per person for a defined benefits package over a specified time regardless of actual care utilisation. This may provide incentives to underprovide care to minimise expenditure or to select low‐risk patients [7]. To offset these incentives and to incentivise the provision of quality care, countries have coupled capitation with performance pay.

This paper presents the main results from case studies of health care purchasing arrangements in Chile, China, Indonesia and South Africa, which involved paying providers of primary care services using capitation with performance pay to improve service quality and health outcomes for chronic conditions. The choice of these four countries was based on global consultations with regional experts to identify middle‐income countries undertaking purchasing initiatives that aimed to improve quality in chronic care and had not been previously documented in the African, American, Western Pacific and Southeast Asian regions of the world. Experts in the four countries completed a structured data collection instrument about the design, implementation and effects based on publicly available information and key informant interviews during 2022 and 2023 [8].

## 3 Implementation

The study settings are described in Table 1. Programs were initiated between 2005 and 2019 and all are ongoing.

**TABLE 1:** Overview of case studies.

Name	Family and Community Health Care Model	National Basic Public Health Services Program (BPHSP)	Chronic Disease Management (disease‐specific) Program (PROLANIS)	Value Care Team (VCT) model
Country, region	Chile, La Pintana (Santiago)	People's Republic of China, nationwide	Indonesia, nationwide	South Africa, Gauteng province, Pretoria North
Type of payment model	Risk‐adjusted capitation with performance payments	Capitation with performance payments	Capitation with performance payments	Risk‐adjusted capitation with performance payments
Prior payment model	Capitation	FFS	Capitation	FFS
Year initiated	2005–2012	2009	2019	2019
Main goal of the programme	To provide access to comprehensive care, improve quality of health services	To achieve universal Basic Public Health Services coverage	To improve quality of life for National Health Insurance (*JKN*) participants with type II diabetes and hypertension	To optimise quality of care and decrease expensive hospital‐based care
Secondary service provision goals	People‐centred primary health care based	Equity in capacity of and access to basic public health services	Integration of care for patients with diabetes and hypertension and strengthening the gate‐keeping function of primary care providers	Strengthening care at primary care level and reducing pressure (cost‐savings) at hospitals
Provider Participation	Mandatory for 6 municipal primary health care facilities	Mandatory for over 45,000 public health centres	Mandatory for 22,373 public and private primary care providers	Voluntary for 4 general practitioner (GP) practices including 17 GPs
Population participation and coverage	144,740; mandatory participation of 77% of eligible population	Mandatory participation of 1.4 billion people	948,432; voluntary participation of 12%–14% of the eligible population (2021)	5620; voluntary participation of 21% of the eligible population
Monthly payment per capita	US$ 18.5, inclusive of performance payments accounting for 8% of the primary health care budget (approx. US$ 1.41)	US$ 0.65 (targeted total capitation payment inclusive of 0.05% performance pay in 2022)	US$ 5 amounting to 93% of funding for primary care	US$ 15.0 for good performance, amounting to 25%–33% of a provider's income
Key performance payment terms	Payments linked to salary levels; payments could be withheld but were not done so in practice	Wide variation in payment level and certainty by county; payments could be reduced	Performance is a factor in determining whether the full capitation payment is paid; no withholding	Payment levels based on performance standards set forth; no withholding
Implementing body	Municipality of La Pintana	Ministry of Finance, and provincial, municipal and county governments	National Health Insurance Agency	Private medical insurance (Government Employee Medical Scheme), private management company (PPO serve)
Estimated annual budget	US$ 32.19 million (2021)	US$ 18.4 billion for total capitation payments (2022)	US$ 1 billion for total JKN capitation payments including PROLANIS (2021)	US$ 1 million (2022)

Abbreviation: FFS, fee for service.

The objectives of the Family and Community Health Care Model in Chile are to ensure access, improve quality and increase social participation in six municipal primary care networks. Providers are paid through a combination of risk‐adjusted capitation payments (71% of the total primary care budget), earmarked funds from central government (19%), pay for performance (6%) and municipal allocations (4%) [9]. The base capitation amount is adjusted for differences in income, population age structure, rural areas, difficulty recruiting staff, and geographic isolation to promote equitable resource allocation. The performance bonus is paid directly to the health care workers in the provider networks which meet performance targets.

Capitation with performance payments is also used in China's National Basic Public Health Services Program to ensure equal access to quality basic public health services. The target group is health care staff at over 45,000 public health centres [10]. Initiated in 2009 to address inequities in facility capacity to deliver a basic package of public health services, the programme was expanded in 2019 to 14 service categories that include hypertension, type 2 diabetes, severe mental disorders and tuberculosis. The standard capitation payment was set centrally and increased incrementally over the years, reaching US$ 13.00 in 2022. The central, provincial, municipal and county governments cover the costs of the capitation. During the study period, the contributions were primarily determined by funding capacity; for example, the central government covered up to 80% of total funding for 12 low‐income provinces. The central government recommended that at least 5% of the capitation payment was determined by performance while the decision about the actual share was made at lower administrative levels; this recommendation has since been dropped.

The *Program Pengelolaan Penyakit Kronis* (PROLANIS) or Chronic Disease Management Program in Indonesia was designed to promote active management of type 2 diabetes and hypertension patients enrolled in Indonesia's national health insurance (Jaminan Kesehatan Nasional; JKN). Initiated in 2014, PROLANIS aims to address poor compliance to medication, patients lost to follow‐up, weak linkages between the primary care team and patients, and low participation in prevention activities. Over 22,000 public and private primary level facilities and providers participate [11]. During the study period, the monthly capitation payment ranged from US$ 0.25 to 1.08 per registered JKN member depending on facility type and performance. JKN covers 93% of funds to facilities and the remaining 7% is paid based on performance.

The value‐based care pilot in Gauteng, Pretoria, South Africa, uses risk‐adjusted capitation with performance bonuses funded by the Government Employees Medical Scheme (GEMS), a private voluntary insurance scheme for civil servants [12]. The pilot aimed to overcome the limitations of fee‐for‐service including supplier‐induced demand and to control costs by reducing hospital admissions and shifting care to primary settings. Initiated in 2019, 21% (5620) of the eligible population enrolled voluntarily. The setting was selected for the pilot because of its high‐risk population and consequent high hospitalisation costs covered by GEMS. The target for the incentives were 4 general practitioner practices and 17 general practitioners. GEMS contracted a private company to manage the pilot and payment system. The performance payment was based on quality scores at both hospital and primary care levels and was adjusted for patient health risks. Combining the risk‐adjusted capitation payment and the performance pay, monthly provider payments amounted to about US$ 15 per capita, of which about half was performance‐based. The programme has since been scaled up to 2 provinces and over 70,000 people.

In all cases, the focus of system change was to strengthen primary care management of chronic disease patients primarily to reduce pressure on referral hospitals and rein in costs. The programs in Indonesia and China also emphasised equity in patients' access to care, medications and prevention activities. In Chile, performance indicators sought to improve diabetes and hypertension targets. The programme in South Africa did not explicitly target chronic diseases but stressed care coordination across hospital and primary care levels with the dual goal of better clinical management and expenditure control.

## 4 Achievements

Generally, substantive achievements require a number of years of implementation and thus early results may be misleading. Only one of the programs underwent independent evaluation. In Indonesia, Sambodo et al. [13] used a difference‐in‐difference study design to assess early impact on public health centres between 2015 and 2016. Compared to the baseline of 1.2%, the evaluation found a statistically significant 0.58% point increase in the monthly percentage of enrollees contacting a public health centre, against the programme target of 15%, and a statistically significant 1.15% point increase among chronically ill patients compared to the baseline of 1.2%, against a target of 50%. The authors report no significant effect on lowering referral rates to hospitals for conditions not requiring specialist care. The authors conclude that the performance‐based capitation reform was not effective in promoting greater use of primary care.

In Chile, capitation was effective in improving equity in the distribution of resources across municipalities, providing essential resources especially to poorer municipalities. However, nearly all (95%) of the provider networks consistently receive 100% of the pay‐for‐performance bonus, and all health workers receive a 10.3% salary bonus even if the network's performance is poor. As such, the overall effectiveness of the pay‐for‐performance scheme is limited.

In China, the central government reduced its share of performance‐based contributions to 14 mostly low‐ and lower‐middle‐income mainland provinces because of lower‐than‐expected performance, and reallocated funds to 17 other provinces with better performance. Therefore, while good performance was rewarded, the payment scheme was regressive in that the funding reductions were concentrated in lower‐income provinces, which likely further reduced quality of services in those areas.

In South Africa, trend data in 2021 showed mixed findings including shorter length of hospital stays and fewer admissions, but also higher rates of surgical admissions. The small‐scale pilot was likely insufficient to drive changes in provider behaviour.

## 5 Enabling factors

There were important organisational interventions that constituted major enabling factors. These included efforts to train and motivate health care providers in line with the performance goals. New categories of health professionals were trained and integrated into the programs in China and South Africa. Health promotion professionals were trained in Indonesia. In South Africa, care coordinators and practice transformation coaches were trained to introduce a team‐based approach to care provision. In China, provincial governments awarded individual providers for outstanding work. Training materials were also developed for staff to increase their capacity to manage chronic disease patients and alliances between hospitals and primary care facilities were promoted to support these trainings.

Improvements in health information systems were also critical. New information systems were introduced in Indonesia and South Africa. Specific data collection platforms and individual health records were established in China. In Indonesia, communication and referral links were made with pharmacy and laboratory networks to ease access to medicines and diagnostics.

Earmarked additional funding was a factor in Chile. A significant amount of additional earmarked funding came from municipal budgets earmarked for primary health care (PHC) improvements corresponding to 26.8% of the annual budget and dedicated to a specific initiative, such as medication review or immunisations. The performance component represents 11.9% of the annual remuneration of eligible workers if the PHC network meets more than 90% of their service delivery goals (i.e., Performance Tier 1). If the PHC network meets between 75% and 90% of the health goals (i.e., Performance Tier 2), the performance component represents 6.0% of remuneration. The performance component is zero if the network meets < 75% of the goals. Payments could be withheld in principle but were not done so in practice. Providers in the other three countries did not receive additional payments or bonuses but their performance was a factor in determining payment levels. For example, in Indonesia, performance determines whether the full capitation payment is paid, and lower performance results in lower monthly capitation payments.

## 6 Challenges

### 6.1 Patient Enrolment

In Indonesia and South Africa, patient enrolment was voluntary and relatively low. In Indonesia, among JKN members with chronic conditions (approximately 6.8 million people), only about 1 million registered for PROLANIS in 2021 [11]. In South Africa, only about 21% of eligible members enrolled in the programme. As patients may have different needs and incentives to enroll, selective enrolment may affect the patient case‐mix and the delivery of appropriate services. It also complicates programme evaluation.

### 6.2 Provider Enrolment

Provider participation was mandatory in all settings except South Africa. Mandatory participation mitigates the effects of adverse selection due to voluntary provider participation, where smaller groups of motivated providers would join if they have below‐average costs or disenrol if they incur financial losses. It is also more likely to yield generalisable results and drive broader improvements. Mandatory participation may be useful for the implementation of care delivery and payment models that have already shown some potential for improving quality through voluntary provider participation [14].

### 6.3 Quality Metrics

All programs used process measures and clinical outcomes as the basis for the performance payment. Many process measures are controlled by the provider and may reflect the quality of their activities more directly. However, clinical outcomes reflect not only provider performance but also patient compliance and self‐management. Furthermore, most clinical outcomes measures, such as laboratory results, rely on routine health information systems, which makes it difficult to disentangle actual changes in the delivery of care from possible improvements in reporting.

### 6.4 Quality Targets

In China and Indonesia, performance targets were fixed and uniform nationwide, primarily linked to national plans and goals, and success was defined by absolute performance. However, some regions were more challenged than others because of lower human resource capacities. In Indonesia, for instance, the providers felt that the targets were unattainable particularly in remote areas where patient recruitment and access to care was difficult. Relative or progressive quality targets may be more appropriate where wide diversity in providers' capacities exist, particularly in national programs, and may be used to encourage gradual quality improvements over time.

### 6.5 Risk Adjustments

Adjusting performance outcomes for population health and social risks and patients' clinical severity are important to reduce incentives for providers to exclude patients with complex or severe conditions. This was done only in South Africa using details about the patients' morbidity status. In the other settings where no risk‐adjustments were made, performance was more difficult to interpret as they could reflect differences in population health risks or socioeconomic factors.

### 6.6 Payment Certainty

Performance targets in Chile were achieved most of the time, resulting in certainty that the maximum performance bonus would be received. In contrast, in China, there was uncertainty regarding the receipt and level of the performance payments because the central guidance for the allocation was subject to change at lower administrative levels. There was little information available in South Africa and Indonesia about how financial incentives were distributed within a given facility.

### 6.7 Payment Level

Low patient volume, low provider uptake, and/or small‐scale implementation may have resulted in relatively low overall amounts of performance pay in China, Indonesia and South Africa. The relatively small incremental rewards or penalties may not have been sufficient to counter the incentives in the base payment method which produces a larger share of their revenue, similar to the situation in much of Europe [14].

### 6.8 Withholding Payments

Withholding or reducing payments for poor performance was done in China and Indonesia. Combined with no risk adjustment, this may have resulted in selectively enrolling healthier patients more willing to comply with medical advice in Indonesia. The large‐scale payment programs in China and Indonesia that did not risk‐adjust or take into consideration differences in provider capacity across wide geographic regions were regressive because the programs rewarded providers that were already well‐resourced and penalised rural and remote areas. Implementing the commonsense approach of ‘not paying for poor quality’ should be done thoughtfully so as not to disadvantage poorly resourced rural providers. Taking away financial resources from providers who are already disadvantaged makes it more difficult for them to make the necessary changes to improve quality of care, thus undermining the objectives of the programme. In Chile, withholding the maximum bonus was possible but was not done in practice. Also, performance bonuses were calculated as a fixed share of health workers' base salary. Given the inequalities in salary, the incentives may have been less effective for lower‐paid staff even though their role in care coordination was important.

## 7 Discussion

This paper presents critical design and implementation features from four diverse cases studies of capitation combined with performance payments for quality chronic care in primary care. These include voluntary enrolment of providers and patients, lack of risk adjustment for performance targets and outcomes, low certainty and withholding of payments. These design and implementation factors contributed to suboptimal uptake and dampened the overall quality improvement effect of the payment incentives. The performance payment itself was insufficient to drive significant changes in provider behaviour to have measurable quality improvements in chronic care.

## 8 Conclusions

The findings from these four countries may be useful in other settings in the design and implementation of payment methods to promote quality chronic care.

## Author Contributions

S.L.B., I.M., M.R. and L.L. designed the study. N.L., Y.L., Q.L., A.S. and S.A.N. led the data collection, analysis and interpretation in each country. S.L.B., I.M., M.R. and L.L. contributed to the data analysis and interpretation.

## Ethics Statement

Ethical approval for this research was waived by the authors' institutes' IRBs.

## Conflicts of Interest

The authors declare no conflicts of interest.

## Data Availability

The data underlying this article are all incorporated into the article.
